# Multi-Omics Integration Improves Polygenic Risk Prediction for Lipid Traits: A Multi-Ancestry Study in UK Biobank

**DOI:** 10.3390/genes17070840

**Published:** 2026-07-22

**Authors:** Nayang Shan, Yafang Qiu, Lin Hou, Zuoheng Wang

**Affiliations:** 1 School of Statistics and Data Science, Capital University of Economics and Business, Beijing 100070, China; shanny2022@cueb.edu.cn (N.S.); qiuyafang0802@163.com (Y.Q.); 2 Department of Statistics and Data Science, Tsinghua University, Beijing 100084, China; houl@tsinghua.edu.cn; 3 MOE Key Laboratory of Bioinformatics, School of Life Sciences, Tsinghua University, Beijing 100084, China; 4 Department of Biostatistics, Yale School of Public Health, New Haven, CT 06510, USA; 5 Department of Biomedical Informatics & Data Science, Yale School of Medicine, New Haven, CT 06510, USA

**Keywords:** multi-omics integration, risk prediction, polygenic risk score, lipid trait, LASSO regression

## Abstract

**Background:** Polygenic risk scores (PRS) have proven valuable for disease risk prediction, but their predictive utility often remains limited because human traits result from complex interactions between environmental and genetic factors. Blood lipid levels are heritable and clinically important risk factors for cardiovascular disease, yet it remains unclear whether multi-omics integration can enhance lipid trait prediction beyond PRS alone. **Methods:** We first constructed single-omics scores, where gene expression, plasma protein, and plasma/serum metabolite levels were genetically predicted and weighted by effect sizes estimated via LASSO regression. Subsequently, we implemented two integration strategies to develop composite multi-omics risk scores (MoRS): step-MoRS, which integrates single-omics scores using stepwise regression, and Lasso-MoRS, which directly models all predicted features across omics layers using LASSO regression. Both approaches were evaluated across European, South Asian, and African ancestries within the UK Biobank. **Results:** MoRS-based methods consistently demonstrated superior predictive accuracy compared to PRS alone for four lipid traits across diverse populations. Notably, Lasso-MoRS prioritized key biomarkers with predictive utility complementary to genomic data. **Conclusions:** These findings confirm that integrating multi-omics biomarkers with genomic data significantly enhances lipid trait prediction across diverse ancestries, offering biological insights into the molecular regulation of lipid metabolism.

## 1. Introduction

Blood lipid traits, including high-density lipoprotein cholesterol (HDL-C), low-density lipoprotein cholesterol (LDL-C), triglycerides (TG), and total cholesterol (TC), are critical heritable risk factors for cardiovascular disease [1,2,3], a leading cause of mortality across diverse populations [4,5]. Genome-wide association studies (GWAS) have successfully identified tens of thousands of single nucleotide polymorphisms (SNP) associated with lipid traits, shedding new light on their etiologies [2,3,6,7,8,9]. Beyond elucidating genetic architecture, these associations provide a foundation for developing genetic risk prediction models, potentially for clinical usage. Polygenic risk scores (PRSs), calculated as weighted sums of risk alleles, are frequently employed to predict complex traits. However, PRS often suffers from limited predictive power and a lack of mechanistic interpretability [10,11]. These limitations partly arise because complex traits result from interplay between genetic variants and intermediate molecular layers, including transcriptome, proteome, and metabolome [12]. There is an ongoing need to integrate information beyond genomics alone. Thus, we aim to investigate whether multi-omics integration can improve polygenic risk prediction for lipid traits.

Several methods have been developed to combine multiple data types. For instance, transcriptional risk scores (TRS) [13], proteomic risk scores (ProRS) [14,15], and metabolic risk scores (MRS) [16,17] have been constructed using measured biomarker levels to stratify risk for conditions such as Crohn’s disease, dementia, and rheumatoid arthritis, demonstrating that multi-omics integration can significantly improve predictive accuracy. However, a major bottleneck in these approaches is their reliance on individual-level omics data, which is often expensive, scarce, and difficult to collect at scale. To overcome this limitation, recent efforts have shifted toward leveraging genetically predicted molecular features. Specifically, several studies have constructed TRS by aggregating imputed gene expression levels and evaluated their utility either within single tissues [18] or across several tissues [19]. To address tissue-selection bias, we previously constructed single-tissue and multi-tissue TRS and systematically evaluated their predictive accuracy beyond using PRS alone [20]. Another study has explored the portability of polygenic TRS across populations in the UK Biobank [21]. Yet, most existing efforts remain limited to single-omics (primarily transcriptome), leaving the combined predictive power of genetically predicted transcriptome, proteome, and metabolome largely unexplored.

This gap is particularly relevant for lipid traits, where ancestry-specific genetic architectures complicate risk prediction. While trans-ancestry PRS methods have improved LDL-C prediction in diverse groups [3,22], and a recent approach utilizing concordant SNPs has enhanced cross-ancestry transferability [23], these models largely ignore the functional molecular intermediates. Multi-omics integration offers an opportunity not only to enhance risk prediction but also to provide insights into the molecular dynamics underlying lipid metabolism.

In this study, we performed an integrative analysis using genetically predicted multi-omics features to predict lipid traits. Extending our previous work [20], we first constructed single-omics scores by weighting genetically predicted gene expression, plasma protein and metabolite levels. The effect sizes of each feature were estimated via the least absolute shrinkage and selection operator (LASSO) regression [24]. Subsequently, we implemented two integration strategies to develop composite multi-omics risk scores: Step-MoRS, which integrates single-omics scores via stepwise regression, and Lasso-MoRS, which directly models all predicted features across omics layers. We compared the performance of both approaches with traditional PRS methods across EUR, SAS, and AFR ancestries in the UK Biobank for four lipid traits. Together, these analyses enabled simultaneous multi-omics-based risk prediction and mechanistic discovery.

The rest of the article is organized as follows. Section 2 describes the study population, the construction of PRS, TRS, ProRS, MRS, and MoRS, and the metrics for model evaluation and comparison. Section 3 reports the model performance and comparisons across ancestries within the UK Biobank for the four lipid traits. Section 4 interprets the findings in the context of the existing literature and discusses their implications as well as the study limitations. Section 5 concludes the article by summarizing the main findings and contributions and outlining future research directions.

## 2. Materials and Methods

### 2.1. Study Population

**Figure 1:**
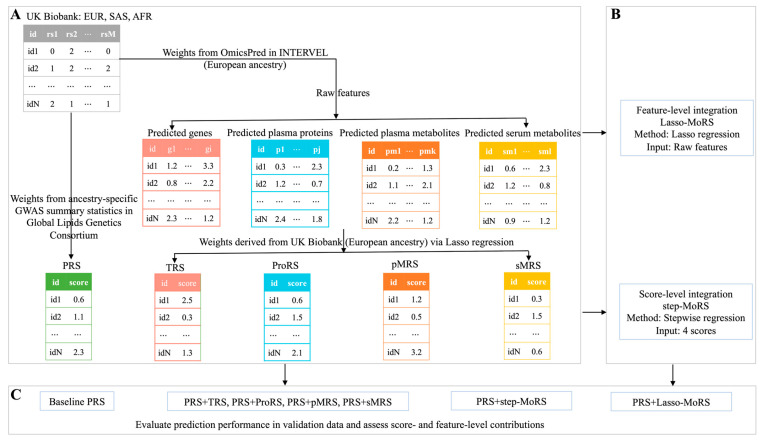
Analysis workflow. (**A**) Data input and construction of single-omics scores. (**B**) Strategies for multi-omics integration. (**C**) Evaluation of predictive accuracy and assessment of score- and feature-level contributions.

The individual genotype and phenotype data were obtained from the UK Biobank (Application number 68672). The UK Biobank obtained ethical approval, and all participants provided informed consent at recruitment. Our analysis was restricted to a set of 407,219 unrelated, high-quality samples [25] and a total of 1,494,152 SNPs in Hapmap3 [26] and Multi-Ethnic Genotyping Arrays [27] that are shared between UK Biobank imputed genotype and GWAS summary statistics. The ancestry information of individuals was obtained from Data-Field 21000. Following ancestry quality control as described previously [21], 349,161 individuals of EUR ancestry, 6779 of SAS ancestry, and 6772 of AFR ancestry were retained. The phenotype measurements of HDL-C, LDL-C, TG, and TC (Data-Fields 30760, 30780, 30870, and 30690) across all available instances were retrieved. Data were aggregated by retaining the first non-missing value, as described previously [21]. HDL-C, LDL-C, and TC were kept on their original scale, while TG was log-transformed in downstream analysis, following the strategy conducted by the Global Lipids Genetics Consortium (GLGC) [3]. We extracted sex (Data-Field 31), age at recruitment (Data-Field 21022), and the first 10 genetic principal components (PCs, Data-field 22009) as covariates. After excluding individuals with missing values for any of four lipid traits or covariates, we had a total of 294,929 EUR, 5721 SAS, 5744 AFR participants for analysis (Figure 1A). The EUR population was randomly split into the training (*n* = 284,929), validation (*n* = 5000), and test (*n* = 5000) sets. For SAS and AFR, samples were split evenly into the validation and test sets. The sample splitting strategy was consistent with that used in the previous study [21].

### 2.2. Construction of PRS

We obtained publicly available large-scale GWAS summary statistics of the four lipid traits for three ancestries from GLGC. The total number of SNPs and median sample size for calculating PRS are displayed in Supplementary Table S1. To establish baseline PRS, we employed two representative methods: the clumping and thresholding (CT) approach [28] and the Bayesian framework PRSCS [29]. The CT method was implemented using PLINK [30] 1.9.0 with a window size of 250 kb and a linkage disequilibrium (LD) $r^{2}$ cutoff of 0.1 in the clumping step. The optimal *p*-value threshold was selected from a range of candidate values (1.0, 5 × 10^−1^, ⋯, 5 × 10^−8^) based on predictive performance in the validation set. PRSCS was implemented using Python 3.12.2. The optimal global shrinkage parameter ϕ was determined from the default candidate values, 1.0, 10^−2^, 10^−4^, and 10^−6^, based on performance in the validation dataset.

### 2.3. Construction of TRS, ProRS, pMRS, sMRS, and MoRS

We calculated genetically predicted levels of gene expression, plasma proteins, and plasma/serum metabolites for all quality-controlled individuals in the UK Biobank. The predicted values were derived as a weighted sum of genotype dosages weighted by effect sizes retrieved from the OmicsPred portal (accession codes OPGS000001-OPGS017227) [31]. The calculations were implemented using the pgsc_calc pipline [32], applying a default variant matching threshold of 0.75 between the weight files and target genomic data. In total, we successfully predicted 13,589 genes, 2678 plasma proteins, 726 plasma metabolites, and 141 serum metabolites.

The form of a single-omics score, a weighted sum of genetically predicted levels of gene expression, plasma proteins, and plasma/serum metabolites, was motivated from our previous TRS work [20]. The weights were estimated via LASSO regression in the training set. We derived single-omics scores, including TRS, ProRS, plasma MRS (pMRS), and serum MRS (sMRS). Subsequently, we implemented two integration strategies to develop composite MoRSs, based on either multiple single-omics scores or features across omics layers (Figure 1B). Step-MoRS, a score-level approach, was developed using forward stepwise regression with Akaike information criterion to identify the optimal combination of pre-calculated single-omics scores. In contrast, Lasso-MoRS, a feature-level approach, was constructed as a linear combination of imputed high-dimensional features across omics layers, weighted by effect sizes estimated via LASSO regression in the training set. Fundamentally, step-MoRS is a low-dimensional integration of multi-omics, whereas Lasso-MoRS is a high-dimensional integration.

### 2.4. Statistical Analyses

To benchmark the performance of the proposed methods, we compared four models (Figure 1C). Model 1 served as the baseline, including age, sex, the first 10 PCs, and PRS. Model 2 included all variables in Model 1 and further incorporated either TRS, ProRS, pMRS, or sMRS individually. Model 3 utilized forward stepwise regression, with Model 1 as the base model, to select the optimal combination of single-omics scores. Model 4 included all variables in Model 1 and further incorporated Lasso-MoRS. Comparison of Models 1 and 2 was used to assess the relative contributions of each single-omics layer. Comparing Models 2 and 3 demonstrated the complementary predictive value of integrating multiple omics. Comparison between Models 3 and 4 evaluated the difference between low-dimensional integration (score level) and high-dimensional integration (feature level).

The EUR training set was employed to derive weights for calculating single-omics scores and Lasso-MoRS. Specifically, we utilized LASSO regression with 5-fold cross-validation to select features and estimate weights. The analysis was performed using the cv.glmnet function in an R package “glmnet” (version 4.1-8) with 5-fold cross-validation, setting nfolds = 5, family = “gaussian”, and type.measure = “mse”. The regularization parameter lambda sequence was automatically generated by glmnet using the default setting. In the 5-fold cross-validation procedure, the input dataset was divided into five folds; in each cross-validation iteration, the model was fitted using four folds and evaluated on the remaining fold. The mean squared error was calculated for each candidate lambda value and averaged across the five folds. We selected the lambda value with the smallest mean cross-validated error. Features with nonzero coefficients at this lambda were retained, and their coefficients were used as the estimated weights. The ancestry-specific validation set was subsequently used for tuning parameters in PRS and estimating the model parameters for single-omics score, step-MoRS, and Lasso-MoRS. The optimized models were then evaluated in an independent ancestry-specific test set.

Predictive performance was measured using adjusted $R^{2}$, controlling for age, sex, and the first 10 PCs. The 95% confidence intervals (CIs) were derived from 1000 bootstrap resamples within the test set, as described previously [33]. To compare model performance, we conducted one-sided paired bootstrap Z tests based on paired differences in the adjusted $R^{2}$. For a given comparison between model A and model B, we calculated the paired bootstrap difference in the adjusted $R^{2}$, $\Delta_{b}=R_{A,b}^{2}-R_{B,b}^{2}$, across 1000 bootstrap replicates. The Z statistic was calculated as $Z=\frac{\hat{\Delta }}{SD(\Delta_{b})},$ where $\hat{\Delta }$ is the observed difference in adjusted $R^{2}$ in the original testing set and $SD(\Delta_{b})$ is the standard deviation of the paired bootstrap differences. One-sided *p* values were calculated as p=1−Φ(Z), testing whether model A achieved higher adjusted $R^{2}$ than model B. We applied this procedure to test whether Lasso-MoRS outperformed step-MoRS, and whether Lasso-MoRS and step-MoRS outperformed the baseline PRS model and the best single-omics model. In all statistical analyses, *p* values less than 0.05 were considered to be significant. All statistical analyses were conducted in R software, version 4.4.1.

To assess whether the gain of our proposed methods was attributable to the incorporation of multi-omics information rather than additional GWAS evidence from the UK Biobank training set, we performed a sensitivity analysis. Specifically, we generated in-house GWAS summary statistics based on 284,929 European participants from the UK Biobank training set. These summary statistics were then used to construct alternative baseline PRS, which were compared with the proposed multi-omics models in the European testing set.

## 3. Results

### 3.1. Applications to the Four Lipid Traits in EUR Ancestry

**Figure 2:**
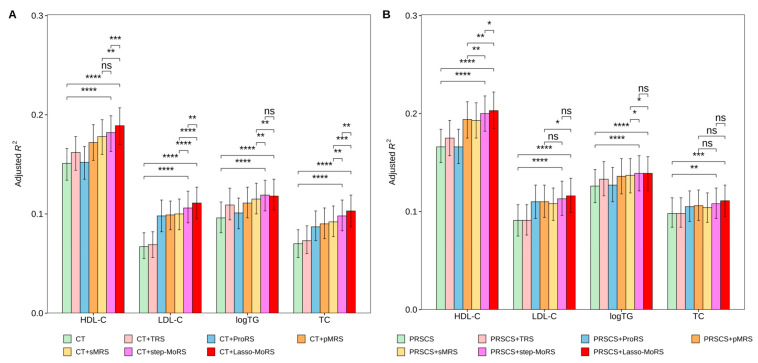
Predictive accuracy across methods for the four lipid traits in EUR. (**A**) Results based on CT-derived PRS. (**B**) Results based on PRSCS-derived PRS. Each colored bar represents the baseline PRS model or the PRS combined with additional omics risk scores: transcriptomic risk score (TRS), proteomic risk score (ProRS), plasma Metabolomic risk score (pMRS), serum Metabolomic risk score (sMRS), multi-omics risk score by stepwise regression (step-MoRS), or multi-omics risk score by Lasso regression (Lasso-MoRS). Bar heights represent the adjusted $R^{2}$ in the test set after accounting for age, sex, and the first 10 PCs. Error bars indicate 95% CIs derived from 1000 bootstrap resamples. *p* values indicate one-sided paired bootstrap Z tests for paired differences in adjusted $R^{2}$ between the models being compared; ns, not significant; *, *p* < 0.05; **, *p* < 0.01; ***, *p* < 0.001; ****, *p* < 0.0001.

We evaluated the proposed MoRS framework with two PRS methods, CT and PRSCS, across the four lipid traits in the EUR population in the UK Biobank (Figure 2, Supplementary Tables S2 and S3). MoRS-based methods consistently outperformed the baseline PRS approaches across all traits. Both step-MoRS and Lasso-MoRS achieved substantial gains in predictive accuracy. Specifically, compared to the CT baseline, step-MoRS improved the adjusted $R^{2}$ by 21% (HDL-C), 58% (LDL-C), 24% (TG), and 40% (TC) (Figure 2A). Similar improvements were observed when benchmarking against PRSCS (gains of 10–24%, Figure 2B). Lasso-MoRS increased the adjusted $R^{2}$ by 25–66% relative to CT (Figure 2A) and 10–27% relative to PRSCS (Figure 2B). Consistent with the observed improvements in adjusted $R^{2}$, one-sided paired bootstrap Z tests showed that both Lasso-MoRS and step-MoRS consistently and significantly outperformed the baseline PRS model. Notably, both Lasso-MoRS and step-MoRS achieved higher adjusted $R^{2}$ than all individual single-omics score methods, confirming that complementary predictive signals exist across different omics layers. One-sided paired bootstrap Z tests further showed that Lasso-MoRS and step-MoRS significantly outperformed the best single-omics model in most trait-PRS settings.

To further validate that the observed improvement was attributable to the incorporation of multi-omics information, we additionally constructed the baseline PRS using GWAS summary statistics generated from 284,929 European training samples in the UK Biobank. MoRS-based methods still improved upon the baseline PRS across all traits (Supplementary Tables S4 and S5).

When comparing the two integration strategies, the feature-level Lasso-MoRS generally achieved higher adjusted $R^{2}$ than the score-level step-MoRS, particularly for HDL-C, LDL-C, and TC. This pattern was supported by one-sided paired bootstrap Z tests, with four of six comparisons reaching statistical significance. For TG, both methods showed comparable performance in adjusted R^2^, and Lasso-MoRS did not significantly outperform step-MoRS (*p* = 0.57 in Figure 2A and *p* = 0.43 in Figure 2B). The performance difference between the two integration approaches suggests that aggregating data into a composite score as in step-MoRS may result in some loss of information. Single-omics score is constructed from features selected within each omics layer. However, when integrated across multiple omics, it is expected that the contribution of each feature to trait prediction may change differently in the presence of features from other omics layers. Thus, Lasso-MoRS, a feature-level integration approach that enables simultaneous feature selection across all omics layers and effectively captures cross-omics signals, is expected to perform better in predicting trait values.

Regarding the contribution of specific single-omics scores, pMRS and sMRS were broadly the most predictive for all lipid traits (Supplementary Tables S2 and S3). However, the contribution of TRS and ProRS varied by traits. TRS provided additional predictive gains for HDL-C and TG, with smaller improvements observed for LDL-C or TC (Supplementary Tables S2 and S3). ProRS enhanced the predictive accuracy for LDL-C and TC, whereas it provided negligible improvement for HDL-C and TG (Supplementary Tables S2 and S3). This heterogeneity underscores the trait-specific architecture of omics regulation and highlights the necessity of an integrative approach.

To investigate the underlying architecture of these integrative models, we analyzed their composition across different omics layers (Supplementary Table S6). Notably, more than 80% of features selected in the Lasso-MoRS model were also included in single-omics scores. Further insights were gained from step-MoRS, which modeled the sequential entry of omics layers. Consistent with their individual performance, sMRS was typically the first to enter the models. This was followed by TRS for HDL-C and TG, or ProRS for LDL-C and TC, suggesting the trait-specific importance of these layers as identified in single-omics evaluations. Despite the excellent predictive performance for pMRS alone, it was almost universally the last to enter the step-MoRS models and consistently failed to retain statistical significance across all lipid traits, or was excluded entirely from the final model. These consistent observations in our model training suggested a high degree of information redundancy in metabolomics in the presence of other omics types. Since pMRS and sMRS captured highly correlated metabolic signals [34], the marginal utility of pMRS diminished once the serum-based score was accounted for. Moreover, the serum metabolomics panel primarily consists of lipoprotein subfractions and lipids, whereas the plasma metabolomics panel captures a broader spectrum of circulating small molecules [31]. As a result, sMRS may be more informative about the four lipid traits. More broadly, this observation highlights the limitations of sequential score-level integration, which can be blinded by collinearity between layers. In contrast, Lasso-MoRS employs global feature selection to handle these redundancies, selecting a non-redundant set of biomarkers from across the entire multi-omics spectrum.

**Figure 3:**
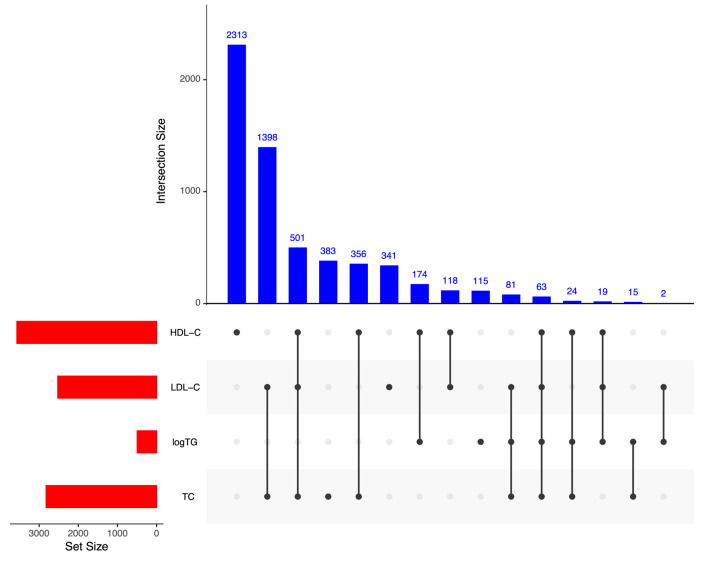
Shared and unique features selected by Lasso-MoRS across four lipid traits. The UpSet plot visualizes the intersections among features selected by Lasso-MoRS for HDL-C, LDL-C, TC, and logTG. The upper bar chart quantifies the number of features unique to each trait or shared across specific combinations of traits. The lower-left bar chart shows the total number of features selected for each lipid trait.

To elucidate the molecular architecture underlying the four lipid traits, we analyzed the features selected by the Lasso-MoRS model, distinguishing between trait-specific and pleiotropic regulatory elements. As visualized in the UpSet plot (Figure 3), the molecular landscape exhibits significant heterogeneity across traits. Among the four traits modeled, HDL-C displayed the greatest biological complexity, with a total of 3568 features, 2313 of which were specific to HDL-C. Furthermore, the model successfully recapitulated the well-established genetic overlap between LDL-C and TC. Previous GWASs have shown that LDL-C and TC share more genetic loci than any other pairs of lipid traits [2]. Consistently, our prediction analysis identified 2043 shared features between LDL-C and TC (Fisher’s exact test: odds ratio = 75.79, *p*-value < 2.2 × 10^−16^), of which 1398 features were exclusive to these two traits, including 1175 genes, 151 proteins, 58 plasma metabolites, and 14 serum metabolites. Most importantly, our intersection analysis pinpointed a pleiotropic core of 63 biomarkers associated with all four lipid traits, representing shared regulators across lipid metabolism. Among these shared signatures, several genes emerged as high-confidence candidates. *BUD13*, located within the pivotal *APOA5*/*A4*/*C3*/*A1* gene cluster, is consistent with previous evidence linking its variants to HDL-C, LDL-C, and TG [35]. *VLDLR* has also been reported to be associated with both LDL-C and TC and to be enriched in pathways including cholesterol metabolism, FXR/RXR activation, lipid transport, and steroid metabolism [2]. *IRS1* was significantly associated with both HDL-C and TG [2]. Notably, several proteins appeared to display pleiotropic effects across the lipid spectrum. GDF15, a stress-responsive cytokine, showed consistent correlations with LDL-C and TC [36], while Cathepsin S was positively associated with TG and negatively associated with HDL-C [37]. Additionally, IL-1Ra levels were associated with lower HDL-C and higher LDL-C, TG, and the TC/HDL-C ratio [38,39,40].

### 3.2. Prediction Performance in SAS and AFR Ancestries

**Figure 4:**
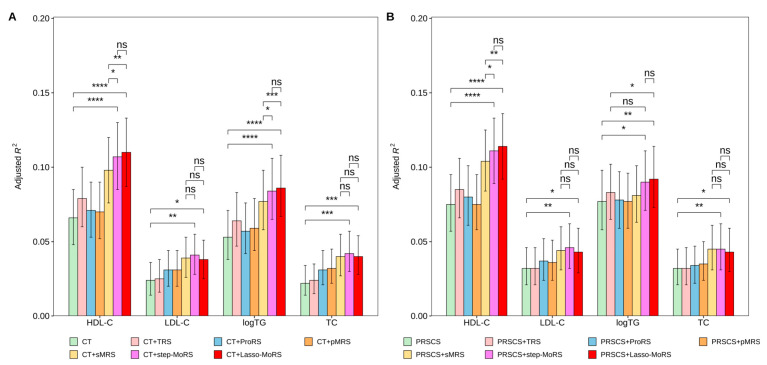
Predictive accuracy across methods for the four lipid traits in SAS. (**A**) Results based on CT-derived PRS. (**B**) Results based on PRSCS-derived PRS. Each colored bar represents the baseline PRS model or the PRS combined with additional omics risk scores: transcriptomic risk score (TRS), proteomic risk score (ProRS), plasma Metabolomic risk score(pMRS), serum Metabolomic risk score (sMRS), multi-omics risk score by stepwise regression (step-MoRS), or multi-omics risk score by Lasso regression (Lasso-MoRS). Bar heights represent the adjusted $R^{2}$ in the test set after accounting for age, sex, and the first 10 PCs. Error bars indicate 95% CIs derived from 1000 bootstrap resamples. *p* values indicate one-sided paired bootstrap Z tests for paired differences in adjusted $R^{2}$ between the models being compared; ns, not significant; *, *p* < 0.05; **, *p* < 0.01; ***, *p* < 0.001; ****, *p* < 0.0001.

**Figure 5:**
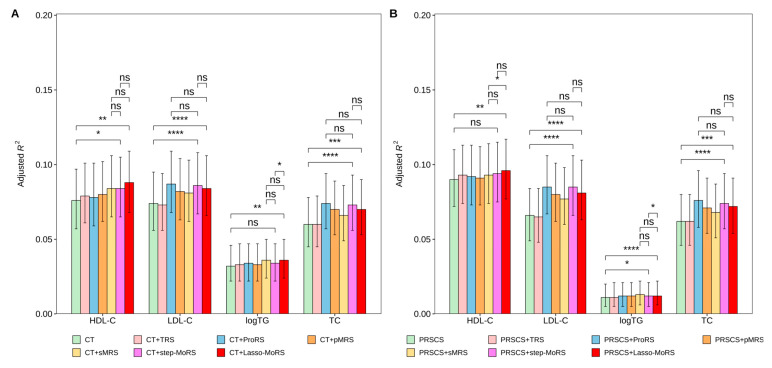
Predictive accuracy across methods for the four lipid traits in AFR. (**A**) Results based on CT-derived PRS. (**B**) Results based on PRSCS-derived PRS. Each colored bar represents the baseline PRS model or the PRS combined with additional omics risk scores: transcriptomic risk score (TRS), proteomic risk score (ProRS), plasma Metabolomic risk score(pMRS), serum Metabolomic risk score (sMRS), multi-omics risk score by stepwise regression (step-MoRS), or multi-omics risk score by Lasso regression (Lasso-MoRS). Bar heights represent the adjusted $R^{2}$ in the test set after accounting for age, sex, and the first 10 PCs. Error bars indicate 95% CIs derived from 1000 bootstrap resamples. *p* values indicate one-sided paired bootstrap Z tests for paired differences in adjusted $R^{2}$ between the models being compared; ns, not significant; *, *p* < 0.05; **, *p* < 0.01; ***, *p* < 0.001; ****, *p* < 0.0001.

To evaluate the cross-ancestry transferability of our methods, we applied the prediction models to four lipid traits in the SAS and AFR populations. PRS were constructed for each cohort using ancestry-specific large-scale GWAS summary statistics from GLGC, whereas the weights used to calculate the single-omics scores and Lasso-MoRS were trained on EUR individuals in the UK Biobank. Models were then validated and tested in independent SAS and AFR cohorts. Despite the use of EUR-trained weights, MoRS-based methods consistently outperformed the benchmark PRS (CT and PRSCS) in both SAS and AFR cohorts (Figure 4 and Figure 5, Supplementary Tables S7–S10), highlighting that multi-omics integration improves predictive performance beyond genomics alone.

The magnitude of improvement varied across ancestries. As observed in EUR, Lasso-MoRS improved prediction accuracy by an average of 40% over CT and 18% over PRSCS, whereas step-MoRS improved accuracy by 36% and 16%, respectively (Supplementary Tables S2 and S3). Larger relative gains were observed in SAS, where Lasso-MoRS outperformed CT and PRSCS by 67% and 35%, respectively, and step-MoRS by 71% and 37% (Supplementary Tables S7 and S8). In AFR, both methods exceeded CT and PRSCS by approximately 15% (Supplementary Tables S9 and S10). Consistent with the EUR results, one-sided paired bootstrap Z tests showed that Lasso-MoRS significantly outperformed the baseline PRS model across all comparisons, whereas step-MoRS showed significant improvement in 12 of 14 comparisons in the SAS and AFR populations. The larger relative improvements in SAS coincided with a lower PRS baseline (Supplementary Tables S7 and S8), likely reflecting the smaller sample sizes of SAS GWAS summary statistics relative to EUR studies (Supplementary Table S1). Accordingly, the integration of transcriptomic, proteomic, and metabolomic signals yielded a greater relative increase in predictive performance. Nevertheless, the absolute adjusted $R^{2}$ values remained lower in SAS and AFR than in EUR, highlighting the importance of further efforts to improve disease risk prediction in non-European populations.

We observed that Lasso-MoRS significantly outperformed step-MoRS only for TG in AFR. Compared with the best single-omics model, both Lasso-MoRS and step-MoRS significantly improved prediction of HDL-C and TG in SAS. In AFR, only Lasso-MoRS showed a significant improvement, limited to HDL-C, whereas step-MoRS showed no significant gain in any comparison. In addition, step-MoRS showed ancestry-specific differences in model composition. The order of feature entry in SAS was broadly consistent with that in EUR, whereas AFR showed a distinct pattern (Supplementary Table S6).

## 4. Discussion

In this study, we developed a multi-omics integration framework to improve prediction of lipid traits across diverse ancestries. By integrating genetically predicted gene expression, plasma proteins, and plasma/serum metabolites, we established a unified strategy for both risk prediction and biological interpretation in settings where individual-level omics data are unavailable.

Our analyses showed that the MoRS-based methods consistently outperformed baseline PRS approaches across populations. The relative improvement was the greatest in SAS, exceeding that observed in EUR, while a modest but still consistent improvement was observed in AFR. Standard PRS often show reduced predictive accuracy in non-European populations because ancestry-specific differences in LD patterns and allele frequencies limit their cross-population transferability [41]. Our framework partially addresses this limitation by integrating genetically predicted transcriptomic, proteomic, and metabolomic information with PRS, even though the omics weights were trained on EUR individuals. Beyond prediction, Lasso-MoRS prioritized several biomarkers associated with lipid regulation, providing insight into the molecular basis of lipid traits. Some of these pleiotropic biomarkers may represent candidate therapeutic targets [42] and could also be leveraged to improve polygenic prediction [43,44,45].

The similarity in model composition between EUR and SAS, together with the divergence observed in AFR, is consistent with established population genetic theory. EUR and SAS populations share more recent common ancestry and more similar LD structures than EUR and AFR populations do, whereas AFR populations are characterized by greater genetic diversity and shorter LD blocks [46]. The strong portability observed in SAS is also supported by previous findings showing that the genetic architecture of lipid metabolism is largely shared between EUR and SAS populations [47].

Taken together, these results suggest that genetically inferred multi-omics features capture lipid-relevant molecular information beyond that captured by conventional PRS. Notably, the presence of both shared and trait-specific selected features further highlights the need for flexible and integrative approaches that can distinguish pleiotropic molecular signals across lipid traits from molecular patterns specific to individual traits. For example, features such as BUD13 and VLDLR were selected across multiple lipid traits, suggesting that they may reflect shared lipid-related signals, whereas some other selected molecular features appeared to be more specific to individual traits.

Several limitations should be acknowledged. First, large-scale non-European omics GWAS summary statistics remain limited, and the omics prediction models, feature selection, and LASSO coefficients in this study were derived primarily from the European training cohort. Although improved prediction was observed in SAS and AFR populations, the stability and portability of the selected transcriptomic, proteomic, and metabolomic features across ancestries remain uncertain. Therefore, it remains unclear whether these features reflect ancestry-shared biological mechanisms or are partly influenced by European-specific genetic architecture. The availability of larger multi-ancestry cohorts and ancestry-diverse omics GWAS resources will likely improve model portability and generalizability and enable ancestry-specific or trans-ancestry evaluation of selected biomarkers.

Second, our analyses focused on four lipid traits, and the extent to which these findings generalize to other complex traits remains unclear. Third, although we evaluated our omics scores in combination with PRS derived using CT and PRSCS, the framework itself is method-agnostic. The derived TRS, ProRS, and MRS can be readily integrated with either single-ancestry or cross-ancestry PRS methods to assess the incremental predictive value they provide beyond PRS across diverse populations in future studies.

Fourth, regular LASSO was used for feature selection. Although this approach improved predictive performance, Lasso-MoRS retained several thousand molecular features for some lipid traits, which may reduce interpretability and limit future clinical implementation. It remains possible that comparable predictive performance may be achieved using a substantially smaller subset of features, but this was not systematically evaluated in the present study. Future studies could evaluate the trade-off between prediction accuracy and model simplicity. In addition, regular LASSO treats predictors individually, may be less stable in the presence of strong feature correlations, and does not leverage predefined group structures or ordering relationships among features. Methods that incorporate structured penalization, such as grouped LASSO, may be useful when pathway-level, protein-family, and metabolite-class information are available.

Finally, the transcriptomic, proteomic, and metabolomic traits used in this study were genetically predicted rather than directly measured, and prediction uncertainty from these genetically inferred molecular traits could propagate into downstream feature selection and risk prediction. Accordingly, our findings should be interpreted as reflecting genetically inferred molecular profiles rather than directly measured molecular traits. Although the selected transcriptomic, proteomic, and metabolomic features provide biological insights into lipid regulation, systematic pathway or gene-set enrichment analyses were not performed in the current study. Future studies incorporating pathway, gene-set, or network-based analyses may further clarify the biological relevance and mechanistic interpretation of the selected molecular signatures. Functional validation and assessment of their clinical utility are also warranted in future studies.

## 5. Conclusions

Integrating genetically predicted transcriptomic, proteomic, and metabolomic features into MoRS substantially improved prediction beyond traditional PRS for four lipid traits across diverse populations. In particular, Lasso-MoRS significantly outperformed the baseline PRS across traits and populations. These findings support multi-omics integration as a promising strategy to improve the accuracy and equity of genetic prediction across diverse populations. More broadly, this study extends current PRS-based risk prediction approaches by incorporating genetically inferred molecular information from multiple omics layers, thereby contributing to the fields of polygenic risk prediction and multi-omics integration, particularly in settings where individual-level omics data are not available.

Future studies could extend this framework to ancestry-diverse omics GWAS resources and larger multi-ancestry cohorts to improve portability and enable ancestry-specific or trans-ancestry evaluation of selected biomarkers. Further work is also needed to assess the generalizability of MoRS to additional complex traits, evaluate structured penalization approaches such as grouped LASSO to improve interpretability and model parsimony, and incorporate pathway, gene-set, or network-based analyses to clarify the biological relevance of selected molecular features. Finally, validation using directly measured omics data, functional experiments, and clinical utility assessment will be important for determining the translational potential of the proposed framework.

## Data Availability

The genotype and phenotype data can be only accessed from the UK Biobank via the approved application. GWAS summary statistics of four lipid traits for three ancestries from GLGC are available at: http://csg.sph.umich.edu/willer/public/glgc-lipids2021/results/ancestry_specific (accessed on 14 November 2024). GWAS summary statistics used to derive genetically predicted gene expression, plasma proteins, and plasma/serum metabolites are publicly accessible through the OmicsPred portal (www.omicspred.org (accessed on 28 December 2024); accession codes OPGS000001-OPGS017227). The analysis scripts used in this paper are available at GitHub: https://github.com/shanny01/MoRS.

## Supplementary Materials

The following supporting information can be downloaded at https://www.mdpi.com/article/10.3390/genes17070840/s1, Table S1: Total number of SNPs and median sample size of GWAS summary statistics in GLGC. Table S2: Adjusted $R^{2}$ and 95% CIs of different methods based on CT in EUR. Table S3: Adjusted $R^{2}$ and 95% CIs of different methods based on PRSCS in EUR. Table S4: Adjusted $R^{2}$ and 95% CIs of different methods based on CT constructed from UK Biobank training set in EUR. Table S5: Adjusted $R^{2}$ and 95% CIs of different methods based on PRSCS constructed from UK Biobank training set in EUR. Table S6: Model composition across omics layers for the four lipid traits. Table S7: Adjusted $R^{2}$ and 95% CIs of different methods based on CT in SAS. Table S8: Adjusted $R^{2}$ and 95% CIs of different methods based on PRSCS in SAS. Table S9: Adjusted $R^{2}$ and 95% CIs of different methods based on CT in AFR. Table S10: Adjusted $R^{2}$ and 95% CIs of different methods based on PRSCS in AFR.

## Abbreviations

The following abbreviations are used in this manuscript:

PRS	polygenic Risk Score
MoRS	Multi-omics Risk Score
EUR	European
SAS	South Asian
AFR	African
HDL-C	High-Density Lipoprotein Cholesterol
LDL-C	Low-Density Lipoprotein Cholesterol
TG	Triglycerides
TC	Total Cholesterol
GWAS	Genome-wide Association Study
SNP	Single Nucleotide Polymorphism
TRS	Transcriptomic Risk Score
ProRS	Proteomic Risk Score
MRS	Metabolomic Risk Score
LASSO	Least Absolute Shrinkage and Selection Operator
PC	Principal Component
GLGC	Global Lipids Genetics Consortium
CT	Clumping and Thresholding
LD	Linkage Disequilibrium
pMRS	plasma Metabolomic Risk Score
sMRS	serum Metabolomic Risk Score
CI	Confidence Interval
