# Supplementary material for: Multi-Omics Integration Improves Polygenic Risk Prediction for Lipid Traits: A Multi-Ancestry Study in UK Biobank
Source: Genes (Basel). 2026 Jul 22;17(7):840. doi: 10.3390/genes17070840 (PMC13409989; doi:10.3390/genes17070840)
Supplement: Supplementary file 1 [file genes-17-00840-s001.zip › genes-4380617-supplementary.pdf]

Table S1. Total number of SNPs and median sample size of GWAS summary statistics in GLGC.

Phenotypes	Ancestry	Total SNPs	Median Sample Size
HDL-C	EUR	1,494,152	884,132
	SAS	1,489,216	33,953
	AFR	1,494,145	90,804
LDL-C	EUR	1,494,152	840,017
	SAS	1,489,170	33,658
	AFR	1,494,143	87,759
logTG	EUR	1,494,152	860,685
	SAS	1,489,187	34,023
	AFR	1,494,144	89,467
TC	EUR	1,494,152	927,970
	SAS	1,489,231	34,135
	AFR	1,494,145	92,554

Table S2. Adjusted R^2 and 95% CIs of different methods based on CT in EUR.

Methods	HDL-C	LDL-C	logTG	TC
CT	0.151 (0.134, 0.169)	0.067 (0.055, 0.083)	0.096 (0.081, 0.111)	0.070 (0.057, 0.082)
CT+TRS	0.162 (0.144, 0.181)	0.069 (0.056, 0.085)	0.109 (0.093, 0.124)	0.073 (0.061, 0.086)
CT+ProRS	0.152 (0.135, 0.170)	0.098 (0.082, 0.113)	0.101 (0.086, 0.117)	0.087 (0.073, 0.102)
CT+pMRS	0.172 (0.154, 0.192)	0.099 (0.084, 0.115)	0.111 (0.095, 0.127)	0.090 (0.075, 0.105)
CT+sMRS	0.178 (0.161, 0.197)	0.100 (0.084, 0.116)	0.115 (0.099, 0.132)	0.092 (0.078, 0.107)
CT+step-MoRS	0.182 (0.164, 0.201)	0.106 (0.091, 0.123)	0.119 (0.102, 0.136)	0.098 (0.083, 0.113)
CT+Lasso-MoRS	0.189 (0.171, 0.208)	0.111 (0.095, 0.129)	0.118 (0.102, 0.135)	0.103 (0.087, 0.118)

Table S3. Adjusted R^2 and 95% CIs of different methods based on PRSCS in EUR.

Methods	HDL-C	LDL-C	logTG	TC
PRSCS	0.166 (0.150, 0.186)	0.091 (0.076, 0.107)	0.126 (0.108, 0.143)	0.098 (0.083, 0.115)
PRSCS+TRS	0.175 (0.158, 0.195)	0.091 (0.076, 0.107)	0.133 (0.115, 0.151)	0.098 (0.083, 0.115)
PRSCS+ProMR	0.166 (0.150, 0.186)	0.110 (0.092, 0.128)	0.127 (0.109, 0.145)	0.105 (0.090, 0.121)
PRSCS+pMRS	0.194 (0.177, 0.215)	0.110 (0.094, 0.128)	0.136 (0.117, 0.155)	0.106 (0.091, 0.122)
PRSCS+sMRS	0.193 (0.175, 0.214)	0.108 (0.091, 0.126)	0.137 (0.118, 0.155)	0.104 (0.089, 0.120)
PRSCS+step-MoRS	0.200 (0.182, 0.221)	0.113 (0.095, 0.132)	0.139 (0.120, 0.156)	0.108 (0.093, 0.124)
PRSCS+Lasso-MoRS	0.203 (0.186, 0.224)	0.116 (0.099, 0.135)	0.139 (0.120, 0.157)	0.111 (0.095, 0.127)

Table S4. Adjusted R^2 and 95% CIs of different methods based on CT constructed from UK Biobank training set in EUR.

Methods	HDL-C	LDL-C	logTG	TC
CT	0.116 (0.100, 0.133)	0.065 (0.053, 0.079)	0.086 (0.072, 0.099)	0.064 (0.052, 0.078)
CT+TRS	0.139 (0.121, 0.156)	0.066 (0.054, 0.080)	0.097 (0.082, 0.112)	0.066 (0.053, 0.080)
CT+ProRS	0.118 (0.103, 0.136)	0.090 (0.076, 0.107)	0.091 (0.077, 0.106)	0.080 (0.066, 0.096)
CT+pMRS	0.151 (0.134, 0.169)	0.091 (0.077, 0.107)	0.103 (0.088, 0.120)	0.083 (0.069, 0.099)
CT+sMRS	0.155 (0.137, 0.174)	0.090 (0.076, 0.107)	0.108 (0.092, 0.125)	0.082 (0.068, 0.098)
CT+step-MoRS	0.163 (0.144, 0.181)	0.096 (0.080, 0.113)	0.111 (0.096, 0.128)	0.088 (0.074, 0.105)
CT+Lasso-MoRS	0.171 (0.152, 0.190)	0.103 (0.088, 0.121)	0.112 (0.096, 0.129)	0.094 (0.079, 0.110)

Table S5. Adjusted R^2 and 95% CIs of different methods based on PRSCS constructed from UK Biobank training set in EUR.

Methods	HDL-C	LDL-C	logTG	TC
PRSCS	0.167 (0.147, 0.185)	0.095 (0.079, 0.111)	0.118 (0.102, 0.135)	0.092 (0.079, 0.108)
PRSCS+TRS	0.169 (0.150, 0.187)	0.096 (0.080, 0.112)	0.120 (0.104, 0.136)	0.093 (0.079, 0.108)
PRSCS+ProMR	0.167 (0.148, 0.185)	0.098 (0.083, 0.115)	0.118 (0.101, 0.135)	0.094 (0.079, 0.110)
PRSCS+pMRS	0.167 (0.148, 0.185)	0.098 (0.082, 0.115)	0.120 (0.104, 0.137)	0.094 (0.080, 0.110)
PRSCS+sMRS	0.178 (0.159, 0.197)	0.097 (0.080, 0.112)	0.128 (0.112, 0.145)	0.089 (0.075, 0.105)
PRSCS+step-MoRS	0.182 (0.163, 0.201)	0.101 (0.084, 0.117)	0.129 (0.113, 0.146)	0.093 (0.079, 0.110)
PRSCS+Lasso-MoRS	0.187 (0.168, 0.206)	0.107 (0.090, 0.123)	0.131 (0.114, 0.148)	0.099 (0.084, 0.116)

Table S6. Model composition across omics layers for the four lipid traits.

Phenotypes	Omics	Lasso-MoRS	Intersection	single-omics score	step-MoRS					
					EUR		SAS		AFR	
					CT	PRSCS	CT	PRSCS	CT	PRSCS
		Number of features			Order of entry					
HDL-C	genes	3,087	2,920	4,305	2	2	2	2	2	2
	plasma proteins	342	291	500	3	3	3	3	1	1
	plasma metabolites	114	101	497	-	-	-	-	3	-
	serum metabolites	25	25	137	1	1	1	1	-	-
LDL-C	genes	2,102	1,656	2,067	3	-	-	-	-	-
	plasma proteins	283	267	642	2	2	2	2	2	1
	plasma metabolites	109	104	462	4*	3*	-	-	-	-
	serum metabolites	29	29	136	1	1	1	1	1	2
logTG	genes	353	349	3,493	2	2	2	2	-	-
	plasma proteins	80	68	149	3	3	4*	4*	1	-
	plasma metabolites	44	38	463	-	-	3	3	-	1
	serum metabolites	16	16	138	1	1	1	1	-	-
TC	genes	2,363	1,935	2,360	3	4*	3*	3*	-	-
	plasma proteins	305	279	646	2	2	2	2	2	2
	plasma metabolites	121	113	468	4*	3*	-	-	-	-
	serum metabolites	32	31	137	1	1	1	1	1	1

*The corresponding score with p-value > 0.05.

Table S7. Adjusted R^2 and 95% CIs of different methods based on CT in SAS.

Methods	HDL-C	LDL-C	logTG	TC
CT	0.066 (0.051, 0.084)	0.024 (0.014, 0.036)	0.053 (0.039, 0.071)	0.022 (0.013, 0.034)
CT+TRS	0.079 (0.062, 0.097)	0.025 (0.015, 0.037)	0.064 (0.047, 0.083)	0.024 (0.014, 0.036)
CT+ProRS	0.071 (0.053, 0.089)	0.031 (0.020, 0.045)	0.057 (0.041, 0.074)	0.031 (0.020, 0.044)

CT+pMRS	0.070 (0.054, 0.089)	0.031 (0.021, 0.044)	0.059 (0.043, 0.077)	0.032 (0.021, 0.046)
CT+sMRS	0.098 (0.078, 0.12)	0.039 (0.026, 0.054)	0.077 (0.058, 0.098)	0.040 (0.028, 0.056)
CT+step-MoRS	0.107 (0.086, 0.128)	0.041 (0.027, 0.056)	0.084 (0.065, 0.107)	0.042 (0.030, 0.059)
CT+Lasso-MoRS	0.110 (0.089, 0.133)	0.038 (0.025, 0.052)	0.086 (0.068, 0.109)	0.040 (0.028, 0.054)

Table S8. Adjusted R^2 and 95% CIs of different methods based on PRSCS in SAS.

**Methods**	**HDL-C**	**LDL-C**	**logTG**	**TC**
PRSCS	0.075 (0.057, 0.095)	0.032 (0.021, 0.044)	0.077 (0.060, 0.098)	0.032 (0.020, 0.045)
PRSCS+TRS	0.085 (0.065, 0.108)	0.032 (0.021, 0.046)	0.083 (0.066, 0.105)	0.032 (0.021, 0.046)
PRSCS+ProMR	0.080 (0.062, 0.102)	0.037 (0.025, 0.051)	0.078 (0.060, 0.098)	0.034 (0.021, 0.048)
PRSCS+pMRS	0.075 (0.057, 0.096)	0.036 (0.025, 0.051)	0.077 (0.060, 0.097)	0.035 (0.023, 0.049)
PRSCS+sMRS	0.104 (0.083, 0.126)	0.044 (0.031, 0.061)	0.081 (0.064, 0.102)	0.045 (0.030, 0.060)
PRSCS+step-MoRS	0.111 (0.089, 0.134)	0.046 (0.032, 0.063)	0.090 (0.071, 0.111)	0.045 (0.030, 0.060)
PRSCS+Lasso-MoRS	0.114 (0.091, 0.139)	0.043 (0.030, 0.059)	0.092 (0.073, 0.114)	0.043 (0.030, 0.058)

Table S9. Adjusted R^2 and 95% CIs of different methods based on CT in AFR.

**Methods**	**HDL-C**	**LDL-C**	**logTG**	**TC**
CT	0.076 (0.057, 0.095)	0.074 (0.056, 0.**094**)	0.032 (0.021, 0.045)	0.060 (0.045, 0.079)
CT+TRS	0.079 (0.060, 0.098)	0.073 (0.055, 0.093)	0.033 (0.021, 0.046)	0.060 (0.045, 0.078)
CT+ProRS	0.078 (0.059, 0.098)	0.087 (0.068, 0.108)	0.034 (0.022, 0.047)	0.074 (0.057, 0.093)
CT+pMRS	0.080 (0.061, 0.100)	0.082 (0.063, 0.104)	0.033 (0.022, 0.046)	0.070 (0.053, 0.089)
CT+sMRS	0.084 (0.064, 0.104)	0.081 (0.062, 0.103)	0.036 (0.024, 0.049)	0.066 (0.050, 0.085)
CT+step-MoRS	0.084 (0.065, 0.104)	0.086 (0.067, 0.108)	0.034 (0.022, 0.047)	0.073 (0.056, 0.092)
CT+Lasso-MoRS	0.088 (0.068, 0.108)	0.084 (0.065, 0.107)	0.036 (0.024, 0.050)	0.070 (0.053, 0.089)

Table S10. Adjusted R^2 and 95% CIs of different methods based on PRSCS in AFR.

**Methods**	**HDL-C**	**LDL-C**	**logTG**	**TC**
PRSCS	0.090 (0.069, 0.113)	0.066 (0.049, 0.083)	0.011 (0.004, 0.019)	0.062 (0.047, 0.080)
PRSCS+TRS	0.093 (0.072, 0.116)	0.065 (0.048, 0.082)	0.011 (0.005, 0.020)	0.062 (0.047, 0.080)
PRSCS+ProMR	0.092 (0.071, 0.116)	0.085 (0.066, 0.104)	0.012 (0.005, 0.020)	0.076 (0.059, 0.095)
PRSCS+pMRS	0.091 (0.070, 0.115)	0.080 (0.062, 0.099)	0.012 (0.005, 0.020)	0.071 (0.055, 0.091)
PRSCS+sMRS	0.093 (0.072, 0.117)	0.077 (0.059, 0.097)	0.013 (0.006, 0.021)	0.068 (0.052, 0.087)
PRSCS+step-MoRS	0.094 (0.073, 0.119)	0.085 (0.066, 0.105)	0.012 (0.005, 0.020)	0.074 (0.057, 0.094)
PRSCS+Lasso-MoRS	0.096 (0.075, 0.119)	0.081 (0.062, 0.101)	0.012 (0.006, 0.021)	0.072 (0.055, 0.091)
